# Macrocyclic MR contrast agents: evaluation of multiple-organ gadolinium retention in healthy rats

**DOI:** 10.1186/s13244-019-0824-5

**Published:** 2020-02-04

**Authors:** Simona Bussi, Alessandra Coppo, Roberto Celeste, Antonello Fanizzi, Alberto Fringuello Mingo, Andrea Ferraris, Catherine Botteron, Miles A. Kirchin, Fabio Tedoldi, Federico Maisano

**Affiliations:** 1 Bracco Imaging SpA, Bracco Research Centre, Via Ribes 5, 10010 Colleretto Giacosa, TO Italy; 2 Bracco SpA, Via Caduti di Marcinelle 13, 20134 Milano, Italy; 3 Sirius Pathology, 1705 route du Salève, 74350 Cruseilles, France; 4 Bracco Imaging SpA, Via Caduti di Marcinelle 13, 20134 Milano, Italy

**Keywords:** Contrast media, Magnetic resonance imaging, Gadolinium, Pharmacokinetics, Histology, Rats

## Abstract

**Objectives:**

The purpose of this study was to compare Gd levels in rat tissues after cumulative exposure to four commercially available macrocyclic gadolinium-based contrast agents (GBCAs).

**Methods:**

Sixty-five male Sprague-Dawley rats were randomized to four exposure groups (*n* = 15 per group) and one control group (*n* = 5). Animals in each exposure group received 20 GBCA administrations (four per week of ProHance®, Dotarem®, Clariscan™, or Gadovist® for 5 consecutive weeks) at a dose of 0.6 mmol/kg bodyweight. After 28-days’ recovery, animals were sacrificed and tissues harvested for Gd determination by inductively coupled plasma-mass spectroscopy (ICP-MS). Histologic assessment of the kidney tissue was performed for all animals.

**Results:**

Significantly (*p* ≤ 0.005; all evaluations) lower Gd levels were noted with ProHance® than with Dotarem®, Clariscan™, or Gadovist® in all soft tissue organs: 0.144 ± 0.015 nmol/g vs. 0.342 ± 0.045, 0.377 ± 0.042, and 0.292 ± 0.047 nmol/g, respectively, for cerebrum; 0.151 ± 0.039 nmol/g vs. 0.315 ± 0.04, 0.345 ± 0.053, and 0.316 ± 0.040 nmol/g, respectively, for cerebellum; 0.361 ± 0.106 nmol/g vs. 0.685 ± 0.330, 0.823 ± 0.495, and 1.224 ± 0.664 nmol/g, respectively, for liver; 38.6 ± 25.0 nmol/g vs. 172 ± 134, 212 ± 121, and 294 ± 127 nmol/g, respectively, for kidney; and 0.400 ± 0.112 nmol/g vs. 0.660 ± 0.202, 0.688 ± 0.215, and 0.999 ± 0.442 nmol/g, respectively, for skin. No GBCA-induced macroscopic or microscopic findings were noted in the kidneys.

**Conclusions:**

Less Gd is retained in the brain and body tissues of rats 28 days after the last exposure to ProHance® compared to other macrocyclic GBCAs, likely due to unique physico-chemical features that facilitate more rapid and efficient clearance.

## Key points

- Macrocyclic gadolinium-based contrast agents (GBCAs) differ in their propensity for Gd retention in rat tissues and organs
- The level of retained Gd may reflect the ease and rapidity of Gd clearance from tissues and organs after administration
- Macrocyclic GBCAs have no impact on rat kidney tissue histology, up to the tested cumulative dose of 12 mmol/kg

## Introduction

It is well established that trace amounts of gadolinium (Gd) are retained in brain and body tissues following the administration of both linear and macrocyclic gadolinium-based contrast agents (GBCAs) [1, 2]. Although the nature of the retained Gd has still to be elucidated, there is strong evidence that GBCAs enter into brain tissues predominantly in the cerebrospinal fluid (CSF) and that Gd retention occurs following its passage from the perivascular space to the interstitial space as part of the normal glymphatic system [3–7]. This mechanism may explain why Gd is retained in the human brain even in the absence of intracranial abnormalities potentially affecting blood-brain barrier permeability [8].

Whereas early reports focused primarily on differences between linear and macrocyclic GBCAs [9, 10], it is now apparent that there are differences also amongst GBCAs of the same class [11–13]. In a recent comparison of three macrocyclic GBCAs in rodents, Bussi et al. [12] ascribed lower levels of Gd retention in the brain after gadoteridol (ProHance®; Bracco Imaging) to differences in the physico-chemical properties of the GBCAs, postulating that the low molecular weight of gadoteridol, combined with lower viscosity and slightly higher lipophilicity [14] compared to gadobutrol (Gadovist®; Bayer Healthcare) [15] and gadoterate (Dotarem®; Guerbet) [16] may facilitate its more efficient clearance via the glymphatic system.

Recently, a fourth macrocyclic GBCA (Clariscan™, gadoteric acid; GE Healthcare) has become available in some countries. This GBCA has the same formulation as Dotarem® [17] but has never been included in either clinical or non-clinical studies of Gd retention. The aim of our study was to confirm and extend the findings of Bussi et al. [12] by assessing the extent to which Gd is retained in rat brain and body tissues after serial administration not only of Dotarem®, Gadovist®, and ProHance® but also of the newer GBCA Clariscan™. At variance with the study of Bussi et al. [12], we adopted validated analytical methods with lower limits of quantification for liver and skin samples to determine whether significant differences in Gd retention are also seen in these tissues. Additional novel aims were to evaluate microscopically the possible impact of these GBCAs on kidney tissue histology and to assess whether there is any influence of GBCA osmolarity (specifically Gadovist 0.5 M vs. Gadovist 1.0 M) on the levels of Gd retention. Finally, we performed computational studies to determine whether hydration-related parameters of the three macrocyclic molecules correlate with Gd clearance from tissues.

## Materials and methods

We followed the methods of Bussi et al. [12]. The study was performed at Charles River Lyon, France (AAALAC accredited), according to site-specific procedures established by the relevant Quality Assurance Unit. Procedures were conducted according to national and international regulations (L.D. 26/2014; Directive 2010/63/EU), Decree 2013-118 relating to the protection of animals used in scientific experiments described in the Journal Officiel de la République Française on 01 February 2013.

### Animal study

Sixty-five male Sprague-Dawley OFA (SD) rats (Charles River Laboratories, France) aged 6 weeks and weighing 129.0–232.5 g at the start of treatment were utilized. After 8 days of acclimation, the animals were randomized to one of five groups: group A (saline; *n* = 5), group B (ProHance®; *n* = 15), group C (Dotarem®; *n* = 15), group D (Clariscan™; *n* = 15), and group E (Gadovist®; *n* = 15). Gadovist® was diluted 1:1 with water for injection (WFI) prior to injection to achieve a similar concentration (0.5 M) and administered volume to those of the other GBCAs investigated. Animals were housed under controlled conditions at 22 ± 3 °C, > 35% relative humidity and 12 h dark/light cycles. Food pellets (A04C-10; Safe, France) and filtered water from municipal services were provided ad libitum.

Animals in groups B, C, D, and E were administered the following contrast agents, respectively: ProHance®, batch n. V17628, expiry: 06/2020; Dotarem®, batch n. 17GD009B01, expiry: 01/2020; Clariscan™, batch n. 13794731, expiry: 12/2018; Gadovist® batches n. 44617D and 72649A, expiry: 11/2017 and 05/2020, respectively. Administration of GBCA or saline was performed at room temperature at the same time each day (between 8.00 a.m. and 12.00 a.m.) into the lateral vein of the tail at an injection rate of 2 mL/min using a Harvard infusion pump. All animals received saline solution (0.9% w/v NaCl) or the respective GBCA at 1.2 mL/kg bodyweight four times a week for 5 consecutive weeks, for a total cumulative dose of 12 mmol/kg bodyweight. The daily administered dose (0.6 mmol/kg bodyweight) corresponds to a clinical dose of 0.1 mmol/kg bodyweight, based on the extrapolation factor for rats [18]. After the 5-week treatment period, each animal was allowed a recovery period of 28 days (corresponding to approximately 2.5 human years [19, 20]) before sacrifice.

### Observations

During the treatment period, all animals were inspected before and after dosing for any clinical signs or reactions to treatment. During the treatment-free period, all animals were inspected once daily. A full clinical examination was performed pre-test and then weekly during the treatment and treatment-free periods.

### Pathology

At the end of the treatment-free period, all animals were necropsied. The animals were killed by carbon dioxide inhalation and exsanguination. After exsanguination and blood sampling, a complete macroscopic post-mortem examination was performed; abnormal findings, if any, were recorded. Thereafter, each animal was dissected to obtain tissues (cerebrum, cerebellum, liver, right kidney, right femur, and skin) for inductively coupled plasma-mass spectrometry (ICP-MS) determination of gadolinium. A total of 455 tissue/blood samples were collected (65 animals; 6 tissue samples, and 1 blood sample per animal). At the same time, the left kidney was dissected and processed to slides for histopathologic evaluation by an experienced ECVP-qualified pathologist (Charles River Laboratories, France).

### Determination of total gadolinium

All procedures were carried out at Bracco Research Centre (Colleretto Giacosa, Turin, Italy). Blood samples (0.5 mL) were mixed 1:2 with nitric acid (65% w/w, Extrapure, Merck). Cerebrum and cerebellum samples were weighed and freeze-dried, and then suspended in 1 mL of nitric acid. The liver, kidney, and skin samples were weighed, freeze-dried, and ground in a mortar. Approximately 0.2 g of each organ was then weighed and suspended in 1 mL of nitric acid. Femurs were weighed and dissolved in 1 mL of nitric acid. All nitric acid solutions were stored at 4 °C for at least 12 h before digestion. Sample mineralization was performed by subjecting the samples to a wet ashing process (95 min at 180 °C for blood, 110 min at 180 °C for the other organs) in a microwave oven system (MARS-5; CEM Corporation). The mineralized samples were quantitatively transferred to disposable Falcon tubes, diluted to 20 mL with 2% nitric acid, filtered at 0.45 μm, and then analyzed by ICP-MS using validated analytical methods. Internal standardization was performed using ^153^Eu. The calibration blanks, calibration standards, and control standard solutions for each analytical sequence were prepared in 2% nitric acid by dilution of a gadolinium oxide (Gd_2_O_3_) standard solution (1000 μg/mL in 2% HNO_3_, Certipur, Merck).

The lower limit of quantitation (LOQ) for gadolinium was 0.1 nmol/mL for blood, 0.1 nmol/g for cerebrum/cerebellum, 0.6 nmol/g for femur, and 1.5 nmol/g for kidney. The LOQ for each of these tissues was verified for accuracy and precision by spiking in triplicate explanted blank organs from untreated animals (not included in this study) with the corresponding amounts of gadolinium and determining the respective percent recoveries. For liver and skin, the LOQ was determined to be 0.1 nmol/g. For these two tissues, the reported values were extrapolated concentrations for samples in which the signal-to-noise (S/N) ratio was at least 10, with the noise corresponding to the mean tissue signal in saline-treated control animals. These extrapolated values were considered conservative, since both the European Medicines Agency (EMA) [21] and Food and Drug Administration (FDA) [22] recommend that the analyte response at the LOQ is at least five times the zero calibrator.

### Assessment of kidney tissue histology

The left kidney of all animals was dissected and fixed in 10% neutral formalin for slide preparation. All slides were stained with hematoxylin and eosin. If abnormal findings were observed, these were graded using a five-point scale from 1 (minimal/very few/very small) to 5 (massive/very many/very large).

### Computational studies

Computational studies were performed to determine whether the minor structural differences between the macrocyclic GBCA molecules impact hydration-related parameters (hydrophilic surface and solvation enthalpy), which reflect the tendency to form ionic or hydrogen bonds primarily with water, but possibly also with hydrophilic macromolecules of the extracellular matrix in body tissues. The hydrophilic surface is a partition of the space around the molecule that defines regions open to interaction with water molecules; the higher the hydrophilic surface value, the higher the number of interactions. The interactions with water molecules can be of different nature (ion-dipole, hydrogen bonding, van der Waals forces), meaning that different energy (enthalpy) levels are released when the molecule is put in water (hydrated). Calculation of hydrophilic surface and solvation enthalpy for gadoterate, gadobutrol and gadoteridol was based, as starting geometries, on the crystallographic structures of the molecules [23–25]. All ab initio calculations were performed at the restricted Hartree-Fock level with the Gaussian 09 W program [26] using the (1 s-4d, 4f7) effective core pseudopotentials with the 5s4p3d-Gaussian-type orbitals valence basis set for the Gd atom [27]. Full geometry optimization of the starting structures was carried out with the 3-21G basis set for the atoms of the ligands, considering one inner-sphere water molecule (*q* = 1).

On the optimized structures, solvation energy was determined by means of single point energy calculations performed with the 6-31G** basis set for the other atoms of the ligands, both in vacuo and in solution, by adopting the polarizable continuum model as implicit solvation model [28]. Hydrophilic surface area values were calculated at the Molecular Mechanics level on the ab initio optimized structures, by means of Goodford’s GRID algorithm [29], as implemented in the Maestro software package [30].

### Statistical analysis

Gadolinium concentration was expressed as nanomoles per gram wet tissue in the case of cerebrum, cerebellum, liver, femur, kidneys, and skin and as nanomoles per milliliter in the case of blood. The Dixon test [31, 32] was used before formal data analysis to highlight possible anomalous data points. Levene’s test [33] was used to test the equality of variance across groups and Shapiro-Wilk’s test [34] was used to assess the normality of the data distribution in each group. Data with homogeneous variances and normal distribution in all groups were analyzed using ANOVA followed by Dunnett’s test [35]. Data showing non-homogeneous variances or a non-normal distribution in at least one group were analyzed using the Kruskal-Wallis test followed by Wilcoxon’s rank-sum test [35]. All statistical analyses were performed at Charles River Laboratories, using SAS software, version 8.2 (Cary, USA).

## Results

All animals successfully underwent all aspects of the study. No unexpected changes in bodyweight were noted and no adverse signs or symptoms were observed for any animal. No gross pathological tissue changes were noted at sacrifice.

**Fig. 1:**
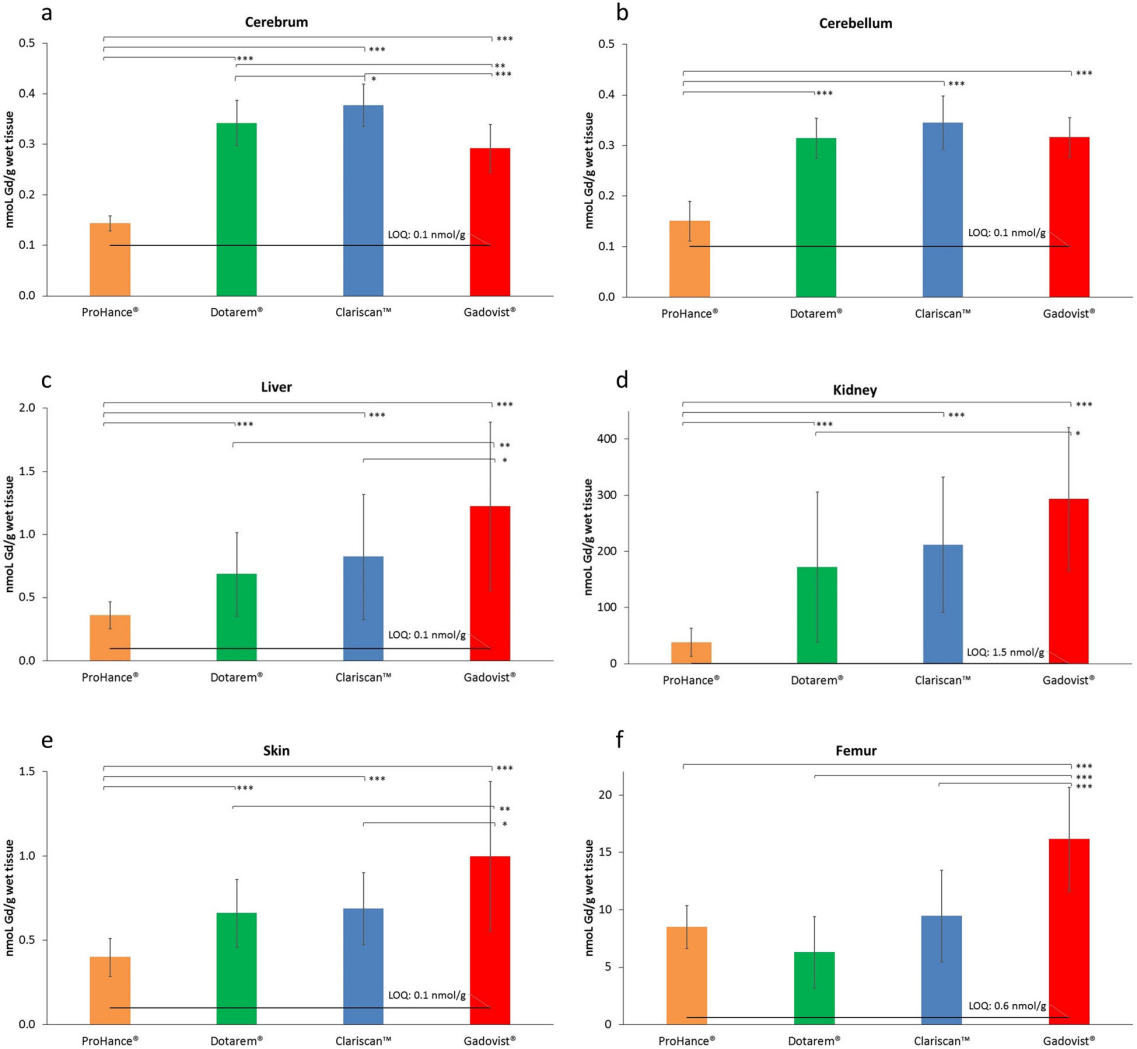
Gadolinium content in the (**a**) cerebrum, (**b**) cerebellum, (**c**) liver, (**d**) kidney, (**e**) skin, and (**f**) femur: Mean values ± SD. The significance of differences between groups is shown as *p ≤ 0.05, **p ≤ 0.01, and ***p ≤ 0.005. Data for blood and for the control group (group A, saline) are not shown because all values were below the LOQ. The error bars represent the standard deviation of measurements within the groups (*n* = 15, except “Dotarem, skin” and “Gadovist, kidney” where *n* = 14)

The mean (± SD) Gd contents across groups and tissue types are presented in Fig. 1 and Table 1. After the 28-day recovery period, the mean Gd levels in the blood of all groups and in all organs from the control group were below the LOQ. Conversely, measurable amounts of Gd were detected in all the tested organs, but with marked differences across the GBCA groups.

**Table 1:** Gadolinium content in the blood, cerebrum, cerebellum, liver, kidney, skin, and femur (mean values ± SD)

Group (ID)	Blood (nmol/mL)	Cerebrum (nmol/g)	Cerebellum (nmol/g)	Liver (nmol/g)	Kidney (right) (nmol/g)	Skin (nmol/g)	Femur (nmol/g)
Control (A)	< LOQ	< LOQ	< LOQ	< LOQ	< LOQ	< LOQ	< LOQ
ProHance (B)	< LOQ^a^	0.144 ± 0.015^b^	0.151 ± 0.039	0.361 ± 0.106	38.6 ± 25.0	0.400 ± 0.112	8.48 ± 1.87
Dotarem (C)	< LOQ	0.342 ± 0.045°°°	0.315 ± 0.040°°°	0.685 ± 0.330°°°	172 ± 134°°°	0.660 ± 0.202°°°	6.28 ± 3.08
Clariscan (D)	< LOQ	0.377 ± 0.042°°^,^*	0.345 ± 0.053°°°	0.823 ± 0.495°°°	212 ± 121°°°	0.688 ± 0.215°°°	9.44 ± 4.01
Gadovist (E)	< LOQ	0.292 ± 0.047°°°^,^**^,###^	0.316 ± 0.040°°°	1.22 ± 0.664°°°^,^**^,#^	294 ± 127°°°^,^*	0.999 ± 0.442°°°^,^**^,#^	16.1 ± 4.51°°°^,^***^,###^

*n* = 5 for control animals; *n* = 15 for all other groups except “Dotarem, skin” and “Gadovist, right kidney” where *n* = 14

Significance vs. group B (ProHance): °°*p* ≤ 0.01, °°°*p* ≤ 0.005; vs. group C (Dotarem): *p ≤ 0.05, **p ≤ 0.01, ***p ≤ 0.005; vs. group D (Clariscan): ^#^*p* ≤ 0.05, ^###^*p* ≤ 0.005

^a^1 out of 15 values > LOQ (0.165)

^b^7 out of 15 values < LOQ

The highest mean Gd levels in the cerebrum and cerebellum were noted after Clariscan followed by Dotarem and Gadovist and finally ProHance. The Gd levels after ProHance were significantly (*p* ≤ 0.005; all evaluations) lower than the levels noted with Dotarem, Clariscan, and Gadovist in both the cerebrum (0.144 ± 0.0147 nmol/g vs. 0.342 ± 0.0448 nmol/g, 0.377 ± 0.0421 nmol/g, and 0.292 ± 0.0473 nmol/g, respectively) and cerebellum (0.151 ± 0.0393 nmol/g vs. 0.315 ± 0.0400 nmol/g, 0.345 ± 0.0525 nmol/g, and 0.316 ± 0.0397 nmol/g, respectively). The Gd level after Gadovist in the cerebrum was higher than after ProHance but significantly lower than after Dotarem (*p* ≤ 0.01) and Clariscan (*p* ≤ 0.005). The difference between Dotarem and Clariscan was not significant (*p* > 0.05) in the cerebellum but was just beyond the limit of significance in the cerebrum (0.342 ± 0.045 nmol/g and 0.377 ± 0.042 nmol/g, respectively; *p* = 0.044).

Similar findings were noted in the right kidney and liver. Significantly lower (*p* ≤ 0.005; all evaluations) Gd levels were noted after ProHance than after Dotarem, Clariscan, and Gadovist in both tissues (38.6 ± 25.0 nmol/g vs. 172 ± 134 nmol/g, 212 ± 121 nmol/g, and 294 ± 127 nmol/g in the right kidney, respectively, and 0.361 ± 0.106 nmol/g vs. 0.685 ± 0.330 nmol/g, 0.823 ± 0.495 nmol/g, and 1.22 ± 0.664 nmol/g in the liver, respectively). Unlike in the cerebrum, the mean Gd level for Gadovist was significantly higher (*p* ≤ 0.05) than that for Dotarem in the right kidney and both Dotarem (*p* ≤ 0.01) and Clariscan (*p* ≤ 0.05) in the liver.

Analogous results were found in the skin. The Gd level after ProHance (0.400 ± 0.112 nmol/g) was significantly lower (*p* ≤ 0.005; all evaluations) than after Dotarem (0.660 ± 0.202 nmol/g), Clariscan (0.688 ± 0.215 nmol/g), and Gadovist (0.999 ± 0.442 nmol/g). The value for Gadovist was significantly higher than that for both Dotarem (*p* ≤ 0.01) and Clariscan (*p* ≤ 0.05).

In the femur, significantly higher (*p* ≤ 0.005, all evaluations) Gd levels were noted with Gadovist than with ProHance, Dotarem, and Clariscan (16.1 ± 4.51 nmol/g vs. 8.48 ± 1.87 nmol/g, 6.28 ± 3.08 nmol/g, and 9.44 ± 4.01 nmol/g, respectively). No significant differences were noted between ProHance, Dotarem, and Clariscan.

### Assessment of kidney tissue histology

**Fig. 2:**
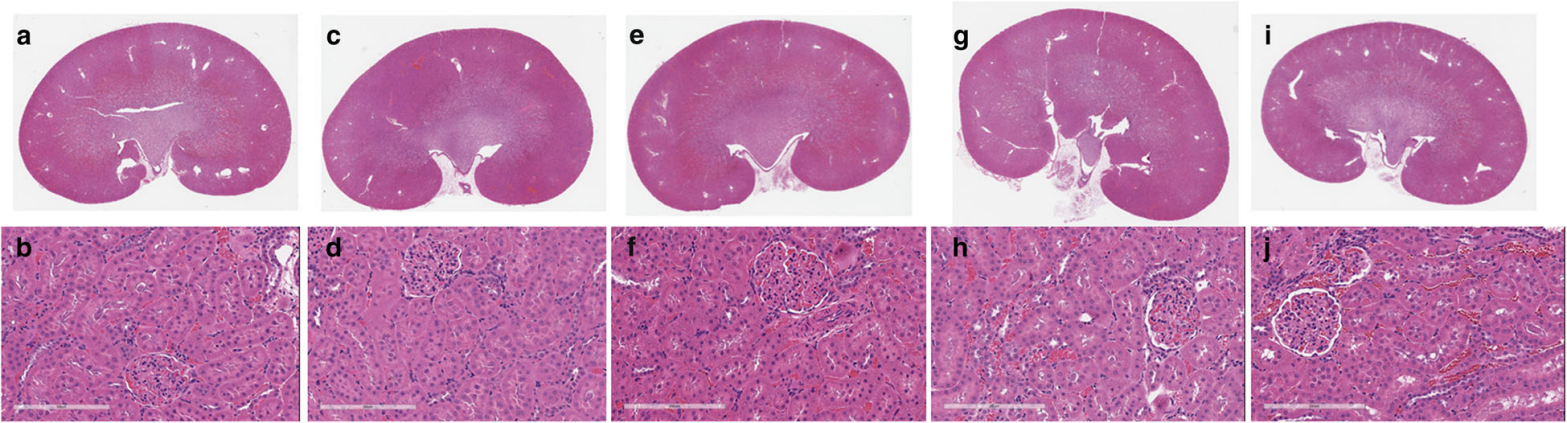
Representative overviews and high magnifications (bar = 200 μm) of kidneys from control animals (**a** and **b**) and from animals administered a total cumulative dose of 12 mmol/kg bodyweight of ProHance (**c** and **d**), Dotarem (**e** and **f**), Clariscan (**g** and **h**), and Gadovist (**i** and **j**). None of the kidneys showed any histologic abnormalities

There were no microscopic observations and no abnormal findings in the left kidney that were considered to be associated with any of the GBCAs (Fig. 2). Any observations were considered incidental and within the range of expected spontaneous changes in rats.

### Computational studies

We calculated the hydrophilic surface and hydration enthalpy for the gadoterate, gadobutrol, and gadoteridol molecules, as indicators of the number and strength of hydrogen bonds and ionic interactions established during dissolution (Table 2). The lowest absolute values in both cases were obtained for gadoteridol.

**Table 2:** Physico-chemical features of the gadoterate, gadobutrol, and gadoteridol molecules

	Gadoterate (Dotarem/Clariscan)	Gadobutrol (Gadovist)	Gadoteridol (ProHance)
*k*_d_ (s^−1^)^a^	7.17 × 10^−14^	1.11 × 10^− 12^	1.15 × 10^− 11^
*t*_½_ (years)^b^	306,549	19,801	1911
Hydrophilic Surface (Å^2^)	491	404	353
Solvation enthalpy (kcal/mol)	˗36	˗12	˗9

^a^*k*_d_ are the rate constants that characterize the dissociation of GBCA complexes in 0.15 M NaCl at 25 °C and pH = 7.4 [43, 44]

^b^*t*_½_ is the half-life (years) of the dissociation reactions of GBCA complexes in 0.15 M NaCl at 25 °C and pH = 7.4 (*t*_½_ = ln2/*k*_d_) [43, 44]

## Discussion

No clinical signs, symptoms, or adverse clinical outcomes have yet been associated with retained Gd in the brain following the repeated administration of any GBCA [36, 37]. Nevertheless, because macrocyclic GBCAs as a class have been associated with lower levels of Gd retention in animal studies [9–11, 13], and with only minor/negligible increases in T1-signal in the dentate nucleus or globus pallidus on unenhanced T1-weighted brain images [38–40] when compared with certain linear GBCAs, the perception is that macrocyclic GBCAs are in some way “safer.” Consequences of this perception have been the suspension by the EMA in Europe of all general-purpose linear agents, and the widespread assumption that all GBCAs within each class are essentially similar and interchangeable. That this is not the case has been highlighted by several recent studies that have shown significant differences amongst GBCAs of the same class in terms of the levels of Gd retained [11–13].

### Tissue Gd concentrations

Our results confirm and extend those of Bussi et al. [12] in showing significantly lower (between 2 and 2.6 times lower) levels of Gd in rat brain (cerebrum and cerebellum) after cumulative administration of ProHance than after equivalent cumulative administration of Dotarem, Clariscan, or Gadovist. These findings have additionally been confirmed by Jost et al. [13] who reported roughly threefold lower levels of Gd in rat cerebellum after ProHance than after Dotarem or Gadovist (0.19 nmol/g vs. 0.54 nmol/g and 0.63 nmol/g, respectively) at 5 weeks, after 8 administrations of each GBCA at 1.8 mmol/kg per injection. Although the retained Gd levels were similar for the three macrocyclic GBCAs at 26 and 52 weeks after the last administration [13], these findings suggest that ProHance is cleared more rapidly than Dotarem or Gadovist, possibly reflecting more efficient migration of the gadoteridol molecule towards the venous and lymphatic vessels in the interstitial space. In this regard, it appears molecular migration is promoted not only by convection, which is unaffected by small differences in otherwise similar molecules, but also by diffusion, which is highly dependent on the intrinsic molecular properties of the GBCAs and their capacity for interaction with the complex surrounding matrix [41].

### Gd clearance from soft tissues

**Fig. 3:**
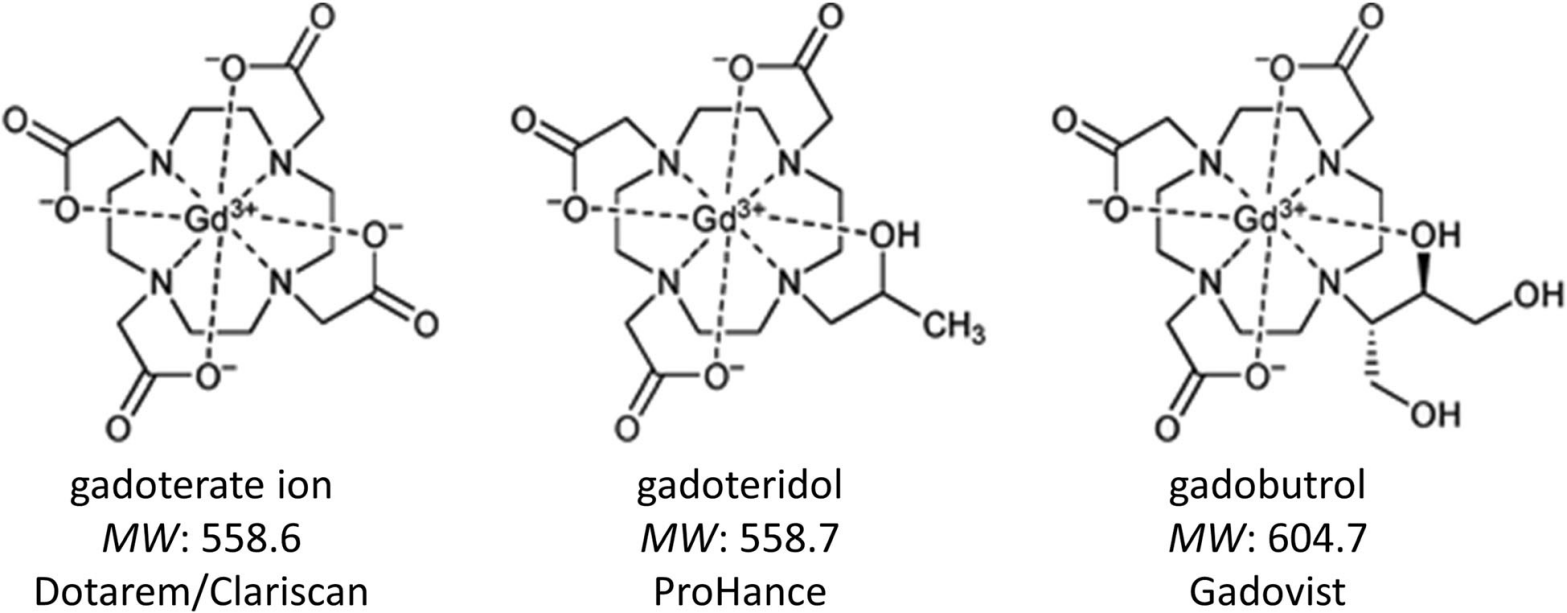
Chemical structures and molecular weights (MW) of macrocyclic GBCAs

As noted in Bussi et al. [12], of the three macrocyclic molecules investigated, gadoteridol has a low molecular weight and other properties that would favor fewer interactions with the surrounding matrix and thus more rapid diffusion and clearance than is the case with gadobutrol and gadoterate. Specifically, since all three molecules are hydrophilic, any interactions with the surrounding matrix that would hinder diffusion leading to slower clearance are likely to occur through hydrogen bonding (e.g., with collagen) and ionic interactions (e.g., with proteoglycan-associated cations). With regards to hydrogen bonding, the gadoteridol molecule carries only one hydroxy group, while the gadobutrol molecule carries three (Fig. 3). As a result, fewer hydrogen bonds are to be expected with gadoteridol than with gadobutrol, resulting in fewer interactions with the surrounding matrix. Likewise, the gadoteridol molecule is non-ionic and thus would be expected to have fewer electrostatic interactions with components of the extracellular matrix than the ionic gadoterate molecule which carries a net negative charge [42]. Our findings for the hydrophilic surface and hydration enthalpy of the three molecules lend support to the initial findings of Bussi et al. [12]. Both these parameters are indicators of the number and strength of hydrogen bonds and ionic interactions established during dissolution in water. The lowest absolute values in both cases were obtained for gadoteridol (Table 2), suggesting that the propensity to establish dipole-dipole interactions with macromolecules rich in hydrogen donor/acceptor groups or ionic charges is lower for gadoteridol than for gadobutrol and gadoterate, respectively.

Of notable interest, we also found significantly lower Gd levels in kidney, liver, and skin after ProHance than after all other GBCAs. Given the strikingly similar profiles of the measured GBCA levels across all soft tissue organs, with ProHance consistently returning lower levels of retained Gd, it is likely the unique physico-chemical properties of the gadoteridol molecule may be relevant not only in the brain [12, 13] but also across all soft tissues.

### Gd levels in bone

Our findings for Gd levels in the femur confirm those of Bussi et al. [12] with significantly higher (*p* ≤ 0.005, all evaluations) levels for Gadovist and non-significantly lower levels for Dotarem relative to ProHance and Clariscan. As in other tissues, the higher Gd levels with Gadovist in bones may reflect increased hydrogen bonding of the gadobutrol molecule with collagen-rich regions [45]. Notably, Lord et al. [46] recently reported Gd levels in the bones of subjects exposed to Gadovist that were not dissimilar to the level reported for a subject exposed to the linear GBCA Omniscan, which is known to lead to greater Gd retention in bones than ProHance [47–49]. The lack of any relevant differences for gadoterate and gadoteridol may be because the extracellular matrix is mineralized in bones, which prevents water flow or diffusion and excludes the contribution of these phenomena to product clearance.

### Histologic evaluation of kidney tissue

Amongst the tissues evaluated, the highest levels of Gd were noted in the kidney, presumably reflecting the fact that this organ is directly involved in GBCA elimination. As in liver and skin, the lowest levels were noted for ProHance followed by Dotarem and Clariscan and finally Gadovist. These findings again confirm those of Bussi et al. [12] and are supported by results from McDonald et al. [11] who found markedly higher median Gd levels in rat kidney at 7 days after 20 administrations of 2.5 mmol/kg Gadovist (555 μg Gd/g of tissue) than after the same dose of ProHance (168 μg Gd/g of tissue).

At variance with our findings, McDonald et al. [11] found more advanced ultrastructural changes in kidneys of ProHance-exposed animals than in kidneys of animals exposed to Gadovist, MultiHance, and Omniscan. These changes included advanced loss of the normal cytoarchitecture of the proximal convoluted tubule, alterations in glomerular structure and filling of Bowman space with matrix and cellular debris, and loss of the outer mitochondrial membrane [11]. Our study revealed no evidence of any macroscopic or microscopic alterations of renal tissues with any GBCA relative to control animals and no evidence of any specific difference with ProHance relative to the other macrocyclic GBCAs. Rather than reflecting a cytotoxic effect of ProHance, the histologic findings described by McDonald et al. [11] are more suggestive of a rat-specific pathology called chronic progressive nephropathy (CPN) [50, 51], and it is possible that the relatively harsh treatment regimen utilized (a cumulative dose of 50 mmol/kg) led to exacerbation of CPN in certain rats. Importantly, CPN has no human counterpart and is not relevant for extrapolation in human risk assessment [52].

### Effects of GBCA formulation properties

Our study differed from that of Bussi et al. [12] in that we included Clariscan for the first time in a Gd retention study and diluted the Gadovist formulation 1:1 with WFI prior to injection. Moreover, thanks to the lower LOQ of the ICP-MS analytical method, we report here also Gd levels in the skin and liver. A comparison of Gd levels across all tissues revealed consistently slightly higher mean levels after Clariscan than after Dotarem although borderline statistical significance (*p* = 0.044) was attained only in the cerebrum. Since the two GBCAs are formulated similarly, the reason for the slight difference is unclear and should be the subject of further study. Concerning the dilution of Gadovist, this was performed to rule out the possibility that the higher Gd levels in brain tissue observed by Bussi et al. [12] were due to a transient osmotic shock of the blood-brain or blood-CSF barriers caused by the higher concentration of the Gadovist formulation. That the brain Gd levels in our study and in that of Bussi et al. [12] were both approximately twofold higher with Gadovist compared to ProHance suggests that transient osmotic shock is not a reason for the higher Gd levels observed with Gadovist.

### Chelate stability

A final consideration concerns the stabilities of the different macrocyclic agents. In all cases, the dissociation half-life exceeds 1900 years at 25 °C in 0.15 M NaCl (Table 2). That none of the agents would dissociate at any point during a patients’ lifetime is suggested by Birka et al. [53] who determined trace levels of intact gadoteridol in the skin of a patient on dialysis who had received ProHance 8 years earlier. That ProHance does not dissociate was also suggested by Gianolio et al. [54] who noted that the amount of residual intact gadoteridol in rat brain after repeat administrations of ProHance corresponded to the total amount of retained Gd. These findings imply that differences between macrocyclic agents in terms of thermodynamic and kinetic stability are irrelevant and have no impact on the levels of retained Gd.

### Study limitations

As with all animal studies, our findings are merely suggestive of the human clinical situation rather than definitive. Healthy animals cannot entirely reproduce the complex physiologic situation in humans and such limitations and restrictions should be borne in mind when decisions are made regarding GBCA usage and safety. For example, whereas Jost et al. [3] reported near-complete clearance of all GBCAs from rat CSF at 24 h after a threefold human equivalent dose of 1.8 mmol/kg bodyweight, Nehra et al. [6] reported significant Gd levels in human CSF at 24 days after a single standard clinical dose of 0.1 mmol/kg intravenous Gadovist. On the other hand, comparative studies can point to inherent differences between GBCAs, which might translate to the human clinical situation even if the precise human physiologic situation differs. Our results confirm those of others [11–13, 47] in noting lower overall Gd levels in animal tissues after repeated exposure to ProHance than after similar repeated exposure to other GBCAs.

A further possible limitation of our study is that Gd levels were determined at only one time point, 28 days, after the last GBCA administration. A recent review [55] suggested that measurements be made at both early (days/weeks) and late (1–2 years) time points after the last administration, since Gd clearance from brain tissue is a relatively slow process. Apart from issues concerning the duration, cost, ethics, and practicality of such studies, a drawback of this approach is the vastly different timescales of the lifespans of rats and humans [19, 20]. Given that 1 rat year corresponds to approximately 30 human years, the impact of senescence-related mechanisms must be considered if Gd levels in rats are assessed after very long time-intervals. In accordance with numerous other studies [54, 56–58] we evaluated Gd retention at a single, specific time point (28 days). Although this does not allow assessment of Gd retention after longer time periods, the results confirm that the elimination curve for gadoteridol from all soft tissue organs is steeper than those of other macrocyclic GBCAs. Moreover, since the measured Gd levels at 28 days after the last administration of ProHance are close to the LOQ, it is highly likely that Gd levels may not be quantifiable by validated analytical methods at later time points.

## Conclusions

In conclusion, our findings confirm and extend those of Bussi et al. [12] in demonstrating considerably lower levels of retained Gd in brain and soft body tissues of rats at 28 days after the administration of ProHance at a total cumulative dose of 12 mmol/kg bodyweight than after equivalent cumulative doses of not only Dotarem and Gadovist but also the newly marketed GBCA, Clariscan™, administered under identical conditions. Furthermore, because of the lower LOQ of the ICP-MS analytical method in this study compared to that of Bussi et al. [12], we were also able to demonstrate significantly lower levels of retained Gd after ProHance in all brain and soft body tissues tested, including the skin and liver. Finally, our findings confirm that GBCA concentration and osmolarity do not influence the amount of retained Gd and show that none of the GBCAs tested have any impact on rat kidney histology, up to the tested cumulative dose.

## Data Availability

The datasets used and/or analyzed during the current study are available from the corresponding author on reasonable request.

## Abbreviations

CPN: Chronic progressive nephropathy
CSF: Cerebrospinal fluid
DOTA: 1,4,7,10-Tetraazacyclododecane-1,4,7,10-tetraacetic acid
ECVP: European College of Veterinary Pathologists
EMA: European Medicines Agency
FDA: Food and Drug Administration
GBCA: Gadolinium-based contrast agent
ICP-MS: Inductively coupled plasma-mass spectrometry
LOQ: Limit of quantitation
WFI: Water for Injection
